# EEG Brain Activity in Dynamic Health Qigong Training: Same Effects for Mental Practice and Physical Training?

**DOI:** 10.3389/fpsyg.2017.00154

**Published:** 2017-02-07

**Authors:** Diana Henz, Wolfgang I. Schöllhorn

**Affiliations:** Institute of Sports Science, University of MainzMainz, Germany

**Keywords:** Health Qigong, dynamic Qigong, mental practice, relaxation, EEG

## Abstract

In recent years, there has been significant uptake of meditation and related relaxation techniques, as a means of alleviating stress and fostering an attentive mind. Several electroencephalogram (EEG) studies have reported changes in spectral band frequencies during Qigong meditation indicating a relaxed state. Much less is reported on effects of brain activation patterns induced by Qigong techniques involving bodily movement. In this study, we tested whether (1) physical Qigong training alters EEG theta and alpha activation, and (2) mental practice induces the same effect as a physical Qigong training. Subjects performed the dynamic Health Qigong technique *Wu Qin Xi* (five animals) physically and by mental practice in a within-subjects design. Experimental conditions were randomized. Two 2-min (eyes-open, eyes-closed) EEG sequences under resting conditions were recorded before and immediately after each 15-min exercise. Analyses of variance were performed for spectral power density data. Increased alpha power was found in posterior regions in mental practice and physical training for eyes-open and eyes-closed conditions. Theta power was increased after mental practice in central areas in eyes-open conditions, decreased in fronto-central areas in eyes-closed conditions. Results suggest that mental, as well as physical Qigong training, increases alpha activity and therefore induces a relaxed state of mind. The observed differences in theta activity indicate different attentional processes in physical and mental Qigong training. No difference in theta activity was obtained in physical and mental Qigong training for eyes-open and eyes-closed resting state. In contrast, mental practice of Qigong entails a high degree of internalized attention that correlates with theta activity, and that is dependent on eyes-open and eyes-closed resting state.

## Introduction

Eastern meditation techniques are a common integral part in everyday life as a means to alleviate working stress, and fostering an attentive mind. One main aim is to enable meditation practitioners to lead a more conscientious style of life. Besides Hindu meditation techniques as different styles of Yoga, Buddhist and Taoist meditation such as Qigong experiences an increasing number of practitioners in the Western hemisphere. Qigong comprises several techniques applied in Traditional Chinese Medicine (TCM) to strengthen physical and mental health. Qigong is commonly divided into static and dynamic forms. Static forms contain meditational techniques whereas dynamic forms afford bodily movements as a tool to direct practitioners’ attention (Tsang et al., 2002; Shinnick, 2006). Research on meditative Qigong practice demonstrates beneficial effects on health (for an overview see Ng and Tsang, 2009). A beneficial influence of Qigong practice on the cardiovascular system, i.e., on blood pressure (Xu, 1994; Lee et al., 2003; Cheung et al., 2005), and electrocardiographic parameters (Lee et al., 2000, 2003) was investigated. Furthermore, Qigong meditation lead to changes in breathing frequency (Sun, 1988). Positive effects of Qigong practice on mental health could be demonstrated in major depression (Tsang et al., 2003; Wang C.W. et al., 2013; Wang F. et al., 2013; Yeung et al., 2013; Yin and Dishman, 2014; Liu et al., 2015; Martinez et al., 2015), anxiety disorders (Lee et al., 2004a,b; Abbott and Lavretsky, 2013; Chan et al., 2013), post-traumatic disorders (Grodin et al., 2008; Kim et al., 2013), in the burnout syndrome (Stenlund et al., 2009, 2012), and in tinnitus (Biesinger et al., 2010). In several studies, a stress alleviating and relaxing effect in healthy subjects (Lee et al., 2005; Posadzki et al., 2010; Terjestam et al., 2010; Glei et al., 2012; Sousa et al., 2012; Hwang et al., 2013; Shim, 2014; Wang et al., 2014) was shown.

An essential research question is how the beneficial effects of Qigong meditation on physical and mental health are mediated by neurophysiological processes. Several studies applying electroencephalography (EEG) and fMRI demonstrated changes in brain activity induced by Qigong meditation. Most studies report increases in theta and alpha activity after Qigong meditation. The first studies on the effect of Qigong meditation on electrical brain activity reported alpha activity predominantly in the anterior brain regions (Wallace, 1970). More differentiated results of effects of meditational Qigong techniques on EEG activity are shown dependent on expertise level. Shifts in alpha activation were observed from posterior to anterior regions during Qigong meditation (Zhang et al., 1988a,b; Jang et al., 2004; Qin et al., 2009). Yang et al. (1994) obtained effects of Zhanzhuang Qigong on brain activity. After 1 year of practice, the alpha activity of the right frontal and right temporal regions increased significantly. The beta index of the right frontal and right temporal regions decreased significantly. A synchronization of brain activity was shown. The effects did not occur after half a year of regular Qigong meditation practice. Therefore, it was assumed to be a gradually adjusting process.

Psychophysiological states of wakefulness and arousal as measured in terms of activation of particular EEG frequency bands are commonly correlated with distinct self-reported experiences of the Qigong state in regular meditators. Mostly, an increase of alpha activity is related to an experience of relaxation and increased well-being. An increase of EEG frontal theta activity is correlated to a self-report of mindfulness, an attentive state which is one of the main aims to reach in Buddhist meditation techniques (for an overview see Tomasino et al., 2014). How are changes in brain activity induced by Qigong meditation correlated to the reported psychophysiological states of relaxation, attentiveness, and attentional processing? In a recent study, EEG alpha-2 activity in posterior right parietal Brodmann areas 5, 7, 31, and 40 during Qigong meditation was demonstrated (Faber et al., 2012). The authors argue that the found patterns of brain activation reflect self-reference, attention and input-centered processing in Qigong meditation. Pan et al. (1994) identified frontal mid-line theta rhythm during the concentrative Qigong state compared to the state of mind reached by non-concentrative Qigong engagement. Shim (2012) found theta activity centering around the frontal lobe parts in Qigong masters and decreased alpha activity compared to beginners. The authors argue that Qigong experts maintained more deeply internalized and relaxed theta activity in the frontal lobe, which reflects an attentive mind. Qigong masters show efficiency in keeping a relaxed and attentive mind around central midline. Lee et al. (1997) investigated effects of ChunDoSunBup Qi-training on brain activity. The Qi-training consisted of acoustic exercises, bodily motion, and meditation. Increases in alpha activity in ChunDoSunBup Qi-training were observed in the occipital regions in eyes-open conditions. The increase in occipital alpha activity was correlated with less self-reported state anxiety. The authors argue that in ChunDoSunBup Qi-training activity of the occipital cortex is reduced and the thalamus is influenced.

On a more structural level, Lehmann et al. (2012) showed reduced functional connectivity between cortical sources in Qigong meditation and reduced functional interdependence between brain regions. These results were interpreted to be a correlate of the reported subjective experience of non-involvement, detachment and letting go, as well as of all-oneness and dissolution of ego borders during Qigong meditation. Cheng et al. (2010) showed an effect of Qigong meditation on prefrontal activity. Practitioners showed in comparison to non-practitioners a significant decrease in deoxyhemoglobin levels suggesting an increase in prefrontal activation during Qigong meditation. Two fMRI studies report changes in brain activity under the state of Qigong during pain exposure in Qigong masters correlating with reduced pain sensation (Chan et al., 2006; Yu et al., 2007). Functional activation in the SII-insula region and other brain areas was reported, whereas a functional suppression under the state of Qigong meditation was observed. Thus, the found functional suppression in brain regions may be responsible for the reduced pain sensation in Qigong masters under the Qigong state.

In conclusion, systematical effects of Qigong meditation on EEG brain activity can be stated with most studies reporting increases in frontal theta and posterior alpha activity as a neurophysiological correlate for a relaxed and attentive mind.

To our knowledge, there are no systematical studies reported in the Western hemisphere on effects on brain activity of dynamic Qigong techniques that afford bodily movement. For instance, the Health Qigong technique *Wu Qin Xi* comprises a consecutive sequence of complex movement configurations. These configurations are symbolic exposures of five animals (tiger, deer, bear, monkey, bird) with each movement sequence performed for several minutes. Practitioners are requested to focus on breathing when performing the movement sequences. According to theoretical assumptions of TCM, *Wu Qin Xi* is an intervention to strengthen especially physical health in general (for an overview see Yang and Wu, 2011). Only a few studies have been reported in the Western hemisphere on the dynamic Qigong technique *Wu Qin Xi* on physical health. Positive effects of *Wu Qin Xi* training are reported on lumbar spinal disease (Yeom et al., 2013; You et al., 2013, 2014; Zhang et al., 2014), blood lipid levels and the antioxidant enzyme activities (Chen, 2011). To date, no studies have been reported in the Western hemisphere on effects of *Wu Qin Xi* on neurophysiological parameters.

In previous experimental studies conducted in our working group, increases in midline fronto-central theta and shifts in alpha activity from posterior to anterior regions over the whole scalp after physical training of the dynamic Qigong technique *Wu Qin Xi* were observed (Henz et al., 2013, 2014, 2015; Henz and Schöllhorn, 2015). From a qualitative point of view, the found brain activation patterns were in line with findings of studies conducted with meditational (static) Qigong. As in studies on meditational Qigong, increases in fronto-central theta and posterior alpha activity were obtained. Thus, a relaxing effect in sense of an evidence-based approach can be stated for the dynamic Qigong technique *Wu Qin Xi*.

As dynamic Qigong affords series of complex bodily movements, practitioners have to invest high effort and attention to learn the new movements. One benefit of that is that directing attention toward the movement execution and kinaesthetic sensations is intended to result in a centered state of internalized concentration. This mechanism plays a key role in mind-body therapies such as dynamic Qigong to draw the attention away from the everyday mind flow to reach an attentive state (for an overview, see Schmalzl et al., 2014).

In the present study, we tested whether the beneficial effects of physical practice of dynamic Qigong on EEG brain activity can be reached by practicing the dynamic Qigong technique *Wu Qin Xi* mentally. From a theoretical point of view, this question is relevant because a large number of brain mapping studies have shown that the same neural areas are activated during either physical or mental simulation of motor actions. The rationale behind is that mental practice with motor content engages areas of the brain that govern movement execution (for an overview see Cicinelli et al., 2006; Sharma and Baron, 2013). This was not only demonstrated for cortical areas such as the supplementary area, the premotor cortex, and the primary motor cortex, but also for subcortical areas such as the basal ganglia and the cerebellum (Lotze et al., 1999; Jeannerod, 2001; Lafleur et al., 2002; Munzert et al., 2008). From a practical point of view, mental Qigong practice becomes relevant for the everyday practitioner in situations when the physical practice of Qigong is impracticable. I.e., when waiting at the train station surrounded by many other passengers it is not possible to practice dynamical Qigong physically due to a limited physical space. Further, this research question is relevant for designs of Qigong courses for elderly persons or patients with bodily impairments. Especially at the beginners’ stage, the movement sequences are practiced many times with several hours of practicing physically leading to increased physical tiredness. Here, the research question arises whether intervals of mental practice sessions would have the same effect on EEG brain activity as a merely physical Qigong practice. Finally, mental Qigong practice becomes relevant especially in persons who do not have the possibility to engage in Qigong training physically, either on the short-term, or on the long-term perspective. For instance, in stroke patients who have a low capability to move or in patients with chronically relapsing diseases of the musculoskeletal system mental practice of *Wu Qin Xi* would be a suitable therapeutic intervention. In achievement sports, when athletes experience phases of immobility due to sports concussions, mental practice of *Wu Qin Xi* Qigong would be an appropriate alternative to physical training.

Recent research has shown that mental practice in the form of motor imagery causes comparable patterns of brain activation as physical training of the same movement. From this, we suppose that mental practice of the dynamic Qigong technique *Wu Qin Xi* leads to comparable effects in EEG brain activation as in physical Qigong training. More precisely, in line with the findings of previous studies on Qigong meditation, and on the dynamic Qigong technique *Wu Qin Xi* on EEG brain activity we hypothesize an increase in EEG midline fronto-central theta and posterior alpha activity when practicing the dynamic Qigong technique *Wu Qin Xi* mentally.

The aims of the present study are as follows:

(1) The analysis of the spontaneous eyes-closed and eyes-open EEG spontaneous activity after training of the dynamic Qigong technique *Wu Qin Xi*.
(2) Comparison of effects of mental practice and physical training of the dynamic Qigong technique *Wu Qin Xi* on the spontaneous eyes-closed and eyes-open EEG brain activity.

## Materials and Methods

### Participants

Twenty-five subjects (mean age 27.9 years, *SD* = 2.91; age range: 19–47; 12 males, 13 females) volunteered in this study. Subjects were recruited from the Qigong workshops at the Institute of Sports Science of the University of Mainz and from sports science courses. Inclusion criteria for the study were participation in a Qigong workshop (30 h of lessons) and at least 1 h practice per week for 1 year. Regular Qigong practice was assessed prior to the experiment by a questionnaire. The subjects were all healthy, and had no current diseases or a history of neurological impairments or intake of medication that may have affected EEG recordings. All subjects were naïve as to the purpose of the current study. All subjects gave written informed consent. The experimental procedures were approved by the local ethics committee at the Johannes Gutenberg University of Mainz, Germany. All experimental procedures were carried out in accordance with the Declaration of Helsinki.

### Experimental Procedure

The subjects were sat comfortably in a dimly-lit isolated room. At each measurement time point, participants began with a resting condition. Spontaneous EEG of the subject was recorded for 2 min for eyes-open, and 2 min for eyes-closed conditions. Then, they were required to perform a 30-min training session. The experiment contained three training conditions: Participants were required to perform the dynamic Qigong technique *Wu Qin Xi* (five animals) physically, and mentally in a within-subjects design. In the mental training condition, subjects were asked to perform the movement sequence mentally from the ego-perspective with imagination of kinaesthetic and visual cues. Further, they were required to apply the same breathing technique in the mental practice condition as in physical training. Additionally, a control condition was tested where subjects were presented a video showing practitioners performing the Qigong exercise *Wu Qin Xi*. All participants were familiar with mental practice of the Qigong technique *Wu Qin Xi*. Experimental conditions were randomized. All training sessions, and the control session were performed with eyes-open. EEG data were obtained during the four resting conditions: (1) pretraining rest, (2) post-Qigong training rest, (3) post-mental Qigong training rest, (4) post-video control rest, which were then used for subsequent analyses.

### EEG Data Acquisition and Analysis

Electroencephalography was recorded through the Micromed Brain quick amplifier and Micromed Brainspy software (Micromed, Venice, Italy). Recordings were taken from nineteen electrodes (Fp1, Fp2, F3, F7, Fz, F4, F8, C3, Cz, C4, T3, T4, P3, P7, Pz, P4, P8, O1, O2) placed according to the Int. Ten to twenty systems with reference to the nose. All electrode impedances were kept at 10 kΩ or below. The EEG signals were continuously recorded and digitized at a sampling rate of 256 Hz. The EEG signal was amplified with a fixed time constant of 0.3 s with a Butterworth second order high-pass filter at 0.5 Hz, and a low-pass filter at 120 Hz (frequency range: 0.5–120 Hz). Electrooculography (EOG) was monitored placed at the medial upper and lateral orbital rim of the right eye (time constant: 0.3 s; high-pass filter: 0.1 Hz; low-pass filter: 120 Hz; frequency range: 0.5–120 Hz).

The spontaneous EEG was recorded for 2 min with eyes-closed, and 2 min eyes-open conditions. Subsequent analyses were performed separately for eyes-closed, and eyes-open conditions. The EEG and EOG signals were visually scored and portions of the data that contained aberrant eye movements, muscle movements of artifacts were removed. The EEG was analyzed and Discrete Fast Fourier Transform was used to obtain the mean power amplitudes in theta (4–7.5 Hz), low-frequency alpha-1 (8–10 Hz), high-frequency alpha-2 (10–12.5 Hz), beta (13–30 Hz), and gamma (30–40 Hz) bands. The ranges of high- and low-frequency alpha bands were defined according to previous studies by Aeschbach et al. (1999) and Cantero et al. (2002).

### Statistical Analyses

A statistical comparison of power of theta, alpha-1, alpha-2, beta, and gamma bands was calculated by repeated-measure analyses of variance (ANOVA) including the within-subject factors as training condition (physical Qigong training, mental Qigong training, video control, baseline rest), condition (eyes-open, eyes-closed), and location (Frontal, Central, Temporal, Parietal, Occipital), followed by Bonferroni corrected *post hoc* tests for further comparisons. $\eta_{P}^{2}$ was calculated to obtain effect sizes. Effects were considered to be statistically significant when the *p*-values were less or equal than 0.05. All data are expressed as the mean ± S.E.

## Results

### Statistical Description: Spontaneous EEG

**FIGURE 1:**
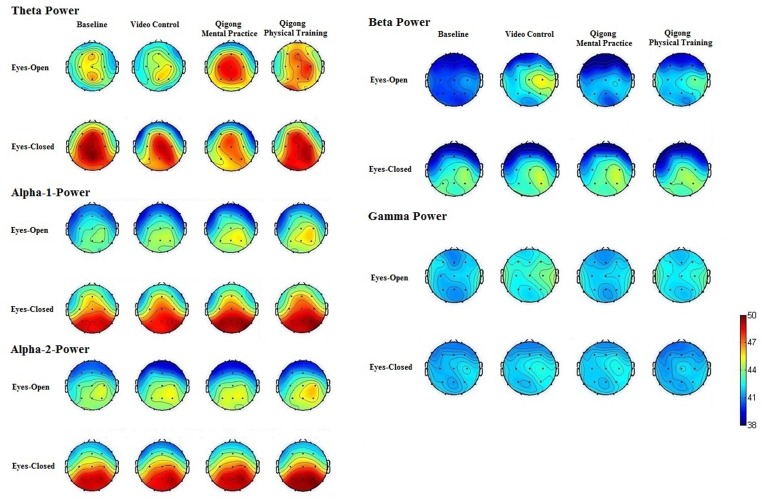
**Spontaneous electroencephalogram (EEG) brain activity at baseline rest, after the video control condition, and after physical and mental Qigong training.** Increased theta and alpha-1 power was obtained after physical and mental Qigong practice. Alpha-2 power was increased after physical Qigong training in the eyes-open condition. In mental Qigong practice, theta power was decreased in the eyes-closed condition.

**Figure 1** shows the mean power spectra for the theta, alpha-1, alpha-2, beta, and gamma band. The ANOVA of theta responses revealed significant differences for training, *F*(3,72) = 3.77, *p* = 0.014, $\eta_{P}^{2}$ = 0.13. *Post hoc* comparisons showed that the spontaneous EEG theta power was significantly increased after physical and mental Qigong training compared to the video control condition, *p* = 0.03, and baseline rest, *p* = 0.01. No significant difference was obtained between physical and mental Qigong practice. The ANOVA of theta responses revealed highly significant differences between conditions (eyes-open, eyes-closed), *F*(1,24) = 8.92, *p* = 0.006, $\eta_{P}^{2}$ = 0.27. The ANOVA of theta responses revealed significant results for training × condition, *F*(3,72) = 3.95, *p* = 0.012, $\eta_{P}^{2}$ = 0.14. *Post hoc* comparisons showed that in mental practice theta activity was decreased in the eyes-closed condition compared to physical training, *p* = 0.007, video control, *p* = 0.021, and baseline rest, *p* = 0.015. The ANOVA of theta responses revealed significant differences between locations, *F*(4,96) = 3.41, *p* = 0.015, $\eta_{P}^{2}$ = 0.12. *Post hoc* comparisons showed that spontaneous EEG theta power was increased at frontal, *p* = 0.017, central, *p* = 0.009, parietal electrodes, *p* = 0.021, and occipital electrodes, *p* = 0.007.

The ANOVA of alpha-1 responses revealed highly significant differences for training, *F*(3,72) = 4.34, *p* = 0.007, $\eta_{P}^{2}$ = 0.15. *Post hoc* comparisons showed that the spontaneous EEG alpha-1 power was significantly increased after physical and mental Qigong training compared to the control condition, *p* < 0.01 each, and baseline rest, *p* < 0.01 each. No significant difference was obtained between physical and mental Qigong practice. The ANOVA of alpha-1 responses revealed highly significant differences between experimental conditions (eyes-open, eyes-closed), *F*(1,24) = 14.088, *p* = 0.001, $\eta_{P}^{2}$ = 0.370. The ANOVA of alpha-1 responses revealed no significant results for the factor training × condition. The ANOVA of alpha-1 responses revealed significant differences between locations, *F*(4,96) = 4.437, *p* = 0.002, $\eta_{P}^{2}$ = 0.156. *Post hoc* comparisons showed that spontaneous EEG alpha-1 power was higher at central, and parietal electrodes than that of frontal, *p* < 0.05 each, temporal, *p* < 0.05 each, and occipital electrodes, *p* < 0.05 each.

The ANOVA of alpha-2 responses revealed significant differences for training, *F*(3,72) = 3.30, *p* = 0.025, $\eta_{P}^{2}$ = 0.12. *Post hoc* comparisons showed that the spontaneous EEG alpha-2 power was significantly higher after physical Qigong training compared to mental practice, *p* = 0.03, video control, *p* = 0.02, and baseline rest, *p* = 0.01. No difference was obtained between mental practice, video control, and baseline rest. The ANOVA of alpha-2 responses revealed highly significant differences between experimental conditions (eyes-open, eyes-closed), *F*(1,24) = 13.38, *p* = 0.012, $\eta_{P}^{2}$ = 0.36. The ANOVA of alpha-2 responses revealed no significant results for training × condition. The ANOVA of alpha-2 responses revealed significant differences between locations, *F*(4,96) = 3.93, *p* = 0.005, $\eta_{P}^{2}$ = 0.14. *Post hoc* comparisons showed that the spontaneous EEG alpha-2 power at central, and parietal electrodes was higher than that of frontal, *p* < 0.05 each, temporal, *p* < 0.05 each, and occipital electrodes, *p* < 0.05 each.

The ANOVA of beta responses revealed significant differences for training, *F*(3,72) = 3.02, *p* = 0.033, $\eta_{P}^{2}$ = 0.11. *Post hoc* comparisons showed that the spontaneous EEG beta power was increased in the video control condition, compared to mental practice, *p* = 0.04, physical training, *p* = 0.02, and resting baseline, *p* = 0.03. Significant differences were found between experimental conditions (eyes-open, eyes-closed), *F*(1,24) = 4.66, *p* = 0.041, $\eta_{P}^{2}$ = 0.16. The ANOVA of beta responses revealed significant results for training × condition, *F*(3,72) = 3.17, *p* = 0.030, $\eta_{P}^{2}$ = 0.12. The ANOVA of beta responses revealed significant differences between locations, *F*(4,96) = 2.74, *p* = 0.033, $\eta_{P}^{2}$ = 0.10. *Post hoc* comparisons showed that the spontaneous EEG beta power at central electrodes was higher than that of frontal, *p* = 0.03, temporal, *p* = 0.04, parietal, *p* = 0.04, and occipital electrodes, *p* = 0.03.

The ANOVA of gamma responses revealed significant differences for training, *F*(3,72) = 3.43, *p* = 0.022, $\eta_{P}^{2}$ = 0.13. *Post hoc* comparisons showed that the spontaneous EEG gamma power was increased in the video control condition, than in mental, *p* = 0.03, and physical Qigong practice, *p* = 0.02, and at baseline rest, *p* = 0.04. Significant differences were found for experimental conditions (eyes-open, eyes-closed), *F*(1,24) = 5.45, *p* = 0.028, $\eta_{P}^{2}$ = 0.19. The ANOVA of gamma responses revealed significant results for training × condition, *F*(3,72) = 3.04, *p* = 0.034, $\eta_{P}^{2}$ = 0.11. *Post hoc* comparisons showed that gamma activity was increased in the video control condition in eyes-open compared to eyes-closed condition, *p* = 0.031. The ANOVA of gamma responses revealed significant differences between locations, *F*(4,96) = 2.83, *p* = 0.029, $\eta_{P}^{2}$ = 0.11. *Post hoc* comparisons showed that spontaneous EEG gamma power at temporal electrodes was increased compared to frontal, *p* = 0.02, central, *p* = 0.03, parietal, *p* = 0.03, and occipital electrodes, *p* = 0.03.

## Discussion

The literature includes several previous investigations on effects of Qigong meditation on EEG brain activity. Most studies report an increase in EEG frontal theta and shift of alpha activity from posterior to anterior regions during and after Qigong meditation. In the present study, we demonstrate an increase of midline fronto-central theta and posterior alpha-1 and alpha-2 activity after practice of the dynamic Qigong technique *Wu Qin Xi*. The finding of increases in midline fronto-central theta and shifts in alpha-1 and alpha-2 activity from posterior to anterior regions after physical training of the dynamic Qigong technique *Wu Qin Xi* was replicated (Henz et al., 2013, 2014, 2015; Henz and Schöllhorn, 2015). Further, our results mirror the findings of previous studies of effects on EEG brain activity after Qigong meditation demonstrating a shift of EEG activity from posterior to anterior regions (Zhang et al., 1988a,b; Yang et al., 1994; Litscher et al., 2001; Jang et al., 2004; Tei et al., 2006a,b, 2009; Qin et al., 2009; Faber et al., 2012). Therefore, a comparable effect for dynamic Qigong on EEG brain activity as found in studies on meditational Qigong can be stated. Training of the dynamic Qigong technique *Wu Qin Xi* induces a relaxed and attentive mind as indicated by an increase in midline fronto-central theta and shifts in alpha activity from posterior to anterior regions. In conclusion, a relaxing effect of the dynamic Qigong technique *Wu Qin Xi* in sense of an evidence-based approach can be stated. Our results indicate that the dynamic Qigong technique *Wu Qin Xi* induces a centered state of mind that has to be distinguished from mind-wandering. Empirical evidence is shown that frontal EEG theta activity is activated in attentional processes and correlates negatively with the default mode network in resting state (Scheeringa et al., 2008).

The highlighted finding of this study is that mental practice of the dynamic Qigong technique *Wu Qin Xi* causes significant modulations of EEG brain activity. Practicing the dynamic Qigong technique *Wu Qin Xi* mentally results in increased fronto-central midline theta activity and increases in alpha-1power in the same intensity as in physical training in eyes open-conditions. Therefore, mental practice of the dynamic Qigong technique *Wu Qin Xi* has the same effect on EEG brain activity as physical training considering the eyes-open condition.

In the present study, the training by condition interaction with respect to changes in the theta band was replicated. In a previous study, it was shown that after training of the dynamic Qigong technique *Wu Qin Xi* theta activity was increased in eyes-open conditions whereas in the eyes-closed condition theta activity was diminished (Henz et al., 2013). In the current study, the same pattern of results with a training × condition interaction was demonstrated. Our results are in line with a study conducted by Aftanas and Golocheikine (2001) who examined EEG brain activity in meditation in eyes-open and eyes-closed conditions. Depending on eyes-open and eyes-closed conditions, different patterns of anterior and midline theta activity occurred. The authors argue that the obtained theta activity reflects internalized attentional processes during meditation that are dependent on eyes-open and eyes-closed states.

The found patterns of brain activations are partially in line with our hypotheses. Our speculation was that based on findings from EEG studies on motor imagery (i.e., Jeannerod, 2006) nearly same effects on EEG brain activity would occur in mental as well as in physical training of the Qigong technique *Wu Qin Xi*. Having a closer look at theta activity, a decrease in mental practice in the eyes-closed condition compared to physical training, and the remaining conditions was observed. Therefore, our results indicate different underlying cognitive and neurophysiological processes in mental and physical *Wu Qin Xi* Qigong training. We hypothesized that different attentional processes during mental and physical *Wu Qin Xi* Qigong training play an essential part that lead to the obtained brain activation patterns. In previous studies on Qigong meditation, frontal and central midline theta activity was associated with internalized attentional processes. For instance, frontal mid-line theta rhythm during the concentrative Qigong state compared to the state of mind reached by non-concentrative Qigong meditation was shown by Pan et al. (1994). In the same manner, Shim (2012) demonstrated frontal theta activity after Qigong meditation in experienced practitioners. In previous studies on Qigong meditation, spontaneous resting EEG was measured with eyes-open. Therefore, no comparison between eyes-open and eyes-closed resting state EEG after Qigong meditation was done although this might allow insights into the underlying attentional processes. For instance, recent studies revealed an association between theta power and switching of involuntary attention from internally directed attention specific to the eyes-closed state to externally directed attention specific to the eyes-open state (Boytsova and Danko, 2009). Specific for meditational states, Aftanas and Golocheikine (2001) demonstrated effects of eyes-open resting state, and eyes-closed resting state on EEG theta activity as a correlate for internalized attentional processes.

One line of argumentation to explain the obtained results for modulations of theta activity dependent on eyes-open and eyes-closed state could be that practicing Qigong physically requires the subjects to strongly direct their attention to the performance of the complex movement sequences and the resulting kinaesthetic sensations as it is conceptualized in mind-body therapies such as Qigong. One main aim is to draw the attention away from the everyday mind flow to reach an attentive state (for an overview, see Schmalzl et al., 2014). As a consequence, a state of deep relaxation is reached in physical training mirroring in increased theta activity in eyes-open as well as eyes-closed conditions.

In physical *Wu Qin Xi* training, many details of the complex movement sequences have to be considered during movement performance, which might lead to a strong internalized attentional processing. From studies on the role of attentional focus during movement performance it is known that an external focus of attention alleviates movement performance, and therefore requires less effort, whereas an internal focus of attention requires more attentional demands. For instance, it has been shown that movement performance benefits from an external focus of attention in gymnastics (Abdollahipour et al., 2015). Several recent studies have provided evidence that movement efficiency, or the physical effort exerted to produce a given performance level or outcome, is also enhanced by an external focus (Zachry et al., 2005; Marchant et al., 2009). Benefits of directing external focus have been found to result in more effective motor performance than those inducing an internal focus by directing attention to the body movements themselves (Totsika and Wulf, 2003; Wulf et al., 2003, 2009; Wulf, 2007). It is argued that focusing on the intended movement effect facilitates the utilization of unconscious or automatic processes, resulting in greater movement ease or fluidity (Wulf et al., 2001; Wulf and Lewthwaite, 2010). Conversely, focusing on one’s own movements leads to a more conscious type of control, thereby constraining the motor system and disrupting automatic control processes (Wulf et al., 2001). It has been shown that relative to an internal focus, an external focus reduces attentional demands and results in the utilization of fast reflexive (automatic) feedback loops (Wulf et al., 2001). Transferring these findings on the physical performance of dynamic Qigong technique *Wu Qin Xi*, induction of internalized attention might be best reached with an attentional demanding complex motor task as in the movement sequences of *Wu Qin Xi*.

From this point of view, an important question arises: does an internal focus of attention during Qigong practice lead to a more demanding type of movement control, and therefore binds more attention which finally results in increased EEG theta and alpha activity? Especially in non-expert practitioners, demanding monitoring processes of movement performance during Qigong practice could result in enhanced stress reduction mirrored by increased EEG theta and alpha activity. We argue that one underlying cognitive mechanism is a working memory load which results from increased motor affordances. From a neurophysiological point of view, frontal theta power has been found to increase with working memory load (Gevins et al., 1997; Krause et al., 2000; Jensen and Tesche, 2002; Onton et al., 2005). Challenging working memory finally results in a loss of a merely executive action control due to limited capacity. Subsequently, practitioners’ attention is drawn away from cognitive engagement in everyday thoughts by a demanding monitoring process. Additionally, a loss of cognitive action control toward a state of non-focusing and non-involvement on the everyday mind flow is one of the main aims in Eastern meditation techniques. Especially in Buddhism-related meditation traditions a mindfulness state is reached by sustained attention on the body. Activations in midline fronto-central lobe structures associated with attentional processes possibly confirm the fundamental role of mindfulness shared by many Buddhist meditations (for an overview see Tomasino et al., 2014). The finding of increased theta activity after physical Qigong training in eyes-closed conditions in our study indicates that internalized attention might be reached more easily when attention and breathing behavior is guided by movements in Qigong training.

A second line of argumentation is that breathing behavior in physical practice underlies a tighter regulation and a stronger forcing due to a coupling to the movement sequences of *Wu Qin Xi* than in mental practice. The breathing technique is strongly determined in the movement sequences of *Wu Qin Xi*, which might lead to the observed increase in low frequencies in the EEG in physical training due to a strong reinforcement by the movement sequences. Recent EEG studies have shown that abdominal breathing techniques lead to increased frontal theta activity (e.g., Yu et al., 2011; Chervin et al., 2012; Park and Park, 2012). Considering breathing as a meditation technique, it was observed that Shaolin Dan Tian Breathing increases EEG frontal theta activity (Chan et al., 2011). The authors argue that the observed increase in frontal theta activity in Shaolin Dan Tian Breathing is a correlate for an attentive mind. Further, several studies have shown that abdominal breathing enhances EEG alpha activity. For instance, Arambula et al. (2001) demonstrated increases in EEG alpha activity in abdominal breathing techniques. Increased alpha band activity with decreased theta band activity was achieved by abdominal breathing during Zen practice (Arita, 2012). Comparing alpha-1 and alpha-2 activity Fumoto et al. (2004) showed increases in alpha-1 activity with disappearance of alpha-2 activity in voluntary abdominal breathing. Therefore, a stronger reinforcement of breathing behavior by movement performance in physical training would be a suitable interpretation for the obtained pattern of EEG brain activity. This might explain a diminished theta activity in mental practice in the eyes-closed condition compared to physical training.

A third line of argumentation considers the role of visual processing during mental practice. Dimitriadis et al. (2015) showed modulations in theta activity as a correlate for mental workload in visual processing. Transferring these findings on mental practice of *Wu Qin Xi*, the decreased theta activity in eyes-closed conditions might reflect attentional demands of visual processing of the movement sequence.

To our knowledge, the present study is the first one that compares effects of mental and physical dynamic Qigong training on EEG brain activity in eyes-open and eyes-closed conditions. From this, we supposed that mental practice of the dynamic Qigong technique *Wu Qin Xi* leads to comparable effects in EEG brain activation than in physical Qigong training. Having a closer look at theta activity in mental practice, a centering around the central areas compared to activation of a broader range of locations after physical training in eyes-open conditions was obtained. From this, we conclude different attentional processes in mental and physical *Wu Qin Xi* Qigong training.

The results of our study have important implications for the design of interventions applying the dynamic Qigong technique *Wu Qin Xi*. Especially in clinical populations who display reduced spontaneous alpha activity as in stress mediated diseases like burnout (see van Luitjelaar et al., 2010), but as well as in anxiety, depression, and bipolar disorders a strong induction of alpha activity by Qigong practice is essential for the therapeutic success of the intervention. With the results of the current study we showed that physical as well as mental training of the Qigong technique *Wu Qin Xi* lead to significant increases in low frequencies in spontaneous EEG. Therefore, mental practice of *Wu Qin Xi* is a suitable alternative therapeutic as to physical dynamic Qigong training.

In the present study, we tested whether the beneficial effects of physical practice of the dynamic Qigong technique on EEG brain activity can be reached by practicing *Wu Qin Xi* mentally. This research question becomes relevant for the everyday practitioner in situations when the physical practice of Qigong is impracticable. Further, this research question is relevant for designs of Qigong courses for elderly persons or patients with bodily impairments. Especially at the beginners’ stage, the movement sequences are practiced many times with several hours of practicing physically leading to increased physical tiredness. Here, the research question arises whether intervals of mental practice sessions would have the same effect on EEG brain activity as a merely physical Qigong practice.

Secondly, mental Qigong practice becomes relevant especially in persons who do not have the possibility to engage in Qigong training physically, either on the short-term, or on the long-term perspective. For instance, in stroke patients who have a low capability to move, mental training of the Qigong technique *Wu Qin Xi* would be an appropriate intervention to induce low frequencies in EEG brain activity and especially stimulate the Mu wave activity in the motor and premotor areas. Recent research has shown that mental practice in the form of motor imagery causes comparable patterns of brain activation as physical training of the movement. The rationale behind is that mental practice with motor content engages areas of the brain that govern movement execution (for an overview see Cicinelli et al., 2006; Sharma and Baron, 2013). Increases in alpha power are reported during mental practice of swimming movements (Beyer et al., 1990). In the same line, changes in EEG alpha oscillations in mental practice of volleyball serves were observed (Stecklow et al., 2010). Transient activations of the M1 area during mental practice are reported (Jeannerod, 1994, 2006; Pfurtscheller et al., 1997; Romero et al., 2000; Munzert et al., 2009). More precisely, inhibition of a movement leads to synchronization in alpha activity whereas preparation, execution and imagery lead to a de-synchronization in sensorimotor areas in the alpha and beta bands (Neuper et al., 2005; Neuper and Pfurtscheller, 2010). In a recent study, it was shown that these specific neuronal circuits are built with increasing experience with a motor task (Nakata et al., 2010). Reiterated engagement of motor areas as in mental *Wu Qin Xi* training is intended to influence brain plasticity phenomena, improving functional outcomes (Cramer et al., 2011; Dimyan and Cohen, 2011; Moisello et al., 2013). Recently, the rehabilitative potential of motor imagery was shown contributing to significantly better motor functional outcomes in sub-acute stroke patients with severe motor impairments (Pichiorri et al., 2015). Further, in patients with chronically relapsing diseases of the musculoskeletal system mental practice of *Wu Qin Xi* would be a suitable therapeutic intervention. Finally, in achievement sports, when athletes experience phases of immobility due to sports concussions, mental practice of *Wu Qin Xi* Qigong would be an appropriate alternative to physical training.

Further research is needed to clarify whether regular mental practice enhances neuroplasticity as shown for physical training of the dynamic Qigong technique *Wu Qin Xi* (Henz et al., 2013) or in meditational Qigong. From studies on the role of expertise level in Qigong practice it was demonstrated that development of a frequency-specific brain excitability is a long-term process. For instance, Shim (2012) demonstrated theta activity centering around the frontal lobe parts (Fp1, Fp2, Fz, F4) in Qigong masters and decreased alpha activity compared to beginners. The authors argue that Qigong experts maintained more deeply internalized and relaxed theta activity in the frontal lobe which reflects an attentive mind. Therefore it is argued that Qigong masters show efficiency in keeping a relaxed and attentive mind around central midline. One further interesting question is whether the same effects would be expected for mental training of the Qigong technique *Wu Qin Xi* in clinical populations with reduced alpha oscillations at resting baseline (see Basar et al., 2012).

## Author Contributions

The authors, DH and WS cooperated on developing the theoretical framework and preparing the manuscript.

## Conflict of Interest Statement

The authors declare that the research was conducted in the absence of any commercial or financial relationships that could be construed as a potential conflict of interest.
